# Sport and self-esteem in people living with HIV: a cross-sectional study

**DOI:** 10.1186/s12879-022-07860-y

**Published:** 2022-11-17

**Authors:** Lucia Grandiere Perez, Clotilde Allavena, Solène Sécher, Sylvain Durand, Antoine Grégoire, Yves-Marie Vandamme, Hikombo Hitoto, Sophie Leautez-Nainville, Christophe Michau, Eric Billaud

**Affiliations:** 1 Service des Maladies Infectieuses et Tropicales, Centre Hospitalier Le Mans, 194 Avenue Rubillard, 72037 Le Mans, France; 2 Service des Maladies Infectieuses et Tropicales, Centre Hospitalier Universitaire Nantes Hôtel-Dieu, 1 Place Alexis Ricordeau, 44093 Nantes Cedex 1, France; 3 COREVIH Pays de la Loire, CHU Nantes Hôtel-Dieu, 1 Place Alexis Ricordeau, 44093 Nantes Cedex 1, France; 4 Département STAPS, EA 4334 Motricité, Interactions, Performance, Le Mans Université, Avenue Olivier Messiaen, 72085 Le Mans Cedex 09, France; 5 Service des Maladies Infectieuses et Tropicales, Centre Hospitalier Universitaire ANGERS, 4 Rue Larrey, 49000 Angers, France; 6 Service de Médecine Post-Urgence, Centre Hospitalier Départemental Vendée, Boulevard Stéphane Moreau, 85925 La Roche Sur Yon Cedex 9, France; 7 Service de Médecine Polyvalente, Centre Hospitalier Saint-Nazaire, 11 Boulevard Georges Charpak BP414, 44600 Saint Nazaire, France

**Keywords:** HIV, Sport, Self-esteem, Fatigue, Pain

## Abstract

**Background:**

In the general population, sport activity is associated with better health and better self-esteem. Among people living with HIV (PLHIV), sport activity could also be associated with better self-esteem. The main objective of our study was to assess the association between sport activity and self-esteem among people living with HIV. The secondary objectives were to evaluate the associations between sport activity with fatigue as well as with pain.

**Methods:**

We performed a cross-sectional observational study among PLHIV in our region (Pays de la Loire in France). Each adult seen in routine HIV care was invited to participate in the study. Participants were invited to fill out self-questionnaires about sport activity, self-esteem, fatigue, and pain. The 2 groups of participants with and without sport activity were compared with a T Student test for self-esteem, fatigue, and pain scales.

**Results:**

Among the 1160 people included in the study, 47% performed sport activity. The self-esteem score was better in the “sporting group” compared with the “non sporting group” (Rosenberg mean scale 32.7 ± 5.1/40 vs 31.9 ± 5 p = 0.01). The Functional Assessment of Chronic Illness Therapy Fatigue scale showed a lower fatigue in the sporting group than in the non-sporting group (mean total score 125 ± 22 vs 118 ± 24 p < 0.0001). The sporting group had a lower mean pain score (1.1 ± 1.8) than the non sporting group (1.4 ± 1.9 p = 0.004).

**Conclusions:**

Among PLHIV in our region, sport activity was associated with better self-esteem, lower fatigue and lower pain. Sport activity should be included in patient care for people living with HIV.

## Background

Sport activity is defined as physical activity to improve physical condition, and set of physical exercises in the form of individual or collective games, practiced by observing certain specific rules (which may give rise to competitions). Among people living with HIV (PLHIV), sport activity is associated with better health, physically and psychologically [1–4]. Self-esteem, defined as the favorable opinion of oneself, is important to the self-management of a chronic illness [5], and is associated with better adherence to anti-retroviral treatment [6]. Sport should improve self-esteem, could be beneficial, and therefore should be included in patient care for patients.

The main objective of our study was to assess the association between sport activity and self-esteem. The secondary objectives were to assess the prevalence of sport activity, and to evaluate the association between sport and fatigue, sport and pain.

## Methods

We performed a cross-sectional study among PLHIV from May to October 2019, in the 5 HIV treatment centers of the Pays de Loire region in France. Each adult seen in routine HIV care was invited to participate in the study. During medical consultation, the practitioner collected informed consent for all subjects. Participants were invited to fill out self-questionnaires about their sport activity (what sports they do (open-ended question), how many times per week, duration of sports sessions). Moreover, they filled out self-questionnaires about self-esteem (Rosenberg scale), fatigue (Functional Assessment of Chronic Illness Therapy Fatigue scale: FACIT-F scale), and pain (analogic pain scale, from 0 to 10). A higher score was associated respectively with higher self-esteem, higher pain and lower fatigue (FACIT-F scales were re-coded such that a higher score indicates lower fatigue). Socio-demographic and clinical data were collected from electronic medical records NADIS^®^. The study was approved by the French national Ethics Committee (Comité de Protection des Personnes).

The 2 groups of participants with and without sport activity were compared with a T Student test on self-esteem, fatigue, and pain scales. The threshold for statistical significance was p < 0.05.

## Results

Among the 4800 PLHIV of the 5 HIV centers of the Pays de Loire region, 1160 answered the self-questionnaire and were included in the study. Participants’ characteristics (Table 1) were similar to the 4800 PLHIV of our region. Among them, 299 were women (26%), 857 men (74%) and 4 transgender (0,3%); the median age was 51 years old and the mean (min–max) age was 50 (age range 18–88). Most of them were born in France (77%) and had a professional activity (61%). Half of the people (49%) declared that they lived alone. Ninety-nine percent of PLHIV were on antiviral treatment; HIV was controlled in most of them: 94% had an undetectable HIV viral load, with a median of CD4 of 698/mm^3^.

**Table 1:** Participants’ characteristics

	All participants n = 1160	No sport activity n = 610	Sport activity n = 550	p value
Gender, *n (%)*				0.33
Female	299 (25.80%)	165 (27.1%)	134 (24.4%)	
Male	857 (73.9%)	444 (72.8%)	413 (75.1%)	
Transgender (M → F)	4 (0.3%)	1	3	
Age (years), *median (IQR)*	51 (58–42)	51 (58–43)	50.5 (59–42)	0.68
BMI, *median (IQR)*	24.1 (27.1–21.7)	24.4 (27.7–21.6)	23.92 (26.3–21.8)	0.22
BMI ≥ 30 kg/m^2^, *n (%)*	139 (12.0%)	86 (14.6%)	53 (10.0%)	0.02
France-native, *n (%)*	893 (77.0%)	474 (77.7%)	419 (76.2%)	0.54
Active employment, *n (%)*	703 (60.6%)	357 (60%)	346 (64.1%)	0.16
Living single, *n (%)*	572 (49.3%)	299 (49.7%)	273 (50.5%)	0.79
CDC stage C, *n (%)*	227 (19.6%)	122 (20%)	105 (19.1%)	0.70
Way of HIV-transmission, *n (%)*				0.28
MSM	572 (49.3%)	295 (48.4%)	277 (50.4%)	
Heterosexual	461 (39.7%)	239 (39.2%)	222 (40.4%)	
Unknown	32 (2.8%)	17 (2.8%)	15 (2.7%)	
Other	95 (8.2%)	59 (9.7%)	36 (6.6%)	
HCV and/or HBV coinfection, *n (%)*	151 (13.0%)	88 (14.4%)	63 (11.5%)	0.13
Duration of HIV infection (years), *median (IQR)*	14.3 (23.5–7.1)	15 (24.4–7.1)	13.6 (22.9–7.1)	0.10
On Current ART, *n (%)*	1146 (98.8%)	601 (98.5%)	545 (99.1%)	0.27
Participants on ART and virologically controlled, *n (%)*	1094 (94.3%)	573 (95.7%)	521 (95.8%)	0.93
Duration of ART (years), *median (IQR)*	11.0 (19.9–5.7)	11.5 (20.5–5.7)	10.6 (19.1–5.7)	0.17
CD4 cells count (/mm^3^), *median (IQR)*	698 (904–510)	705 (910–505)	694 (899–520)	0.82
Nadir CD4 cells count (/mm^3^), *median (IQR)*	261 (387–131)	245 (368–122)	274 (403–145)	0.04

*ART* Antiretroviral therapy, *BMI* body mass index, *CD4* Cluster of differentiation 4, *CDC* centers for disease control, *HCV* hepatitis C infection, *HBV* hepatitis B infection, *HIV* human immunodeficiency virus, *IQR* interquartile range, *MSM *men who have sex with men

Participants’ characteristics of the total population (n = 1160) and each sub-group (with or without sport activity)

Sport activity was performed by almost half of the people (47%): 44% of women, 48% of men and 3 of 4 transgender participants. Jogging or bicycling were the most common sports (45%), followed by strength training (30%) and hiking (27%). Most people (51%) said they did 2 or more sports. Almost half of the subjects with sport activity (46%) did sport 3 times a week or more often.

The self-esteem score was better in the sporting group compared with the non-sporting group (Rosenberg mean scale 32.7 ± 5.1/40 vs 31.9 ± 5/40; p = 0.01). The FACIT-F total score showed lower fatigue in the sporting group than in the non sporting group (125 ± 22 vs 118 ± 24; p < 0.0001). The sporting group had a lower mean pain score (1.1 ± 1.8) than the non-sporting group (1.4 ± 1.9 p = 0.004) (Table 2).

**Table 2:** Results of the self-esteem, fatigue and pain scales

Scales	No sport activity N = 610	Sport activity N = 550	p value (T Student test)
Self-esteem Rosenberg scale, (mean ± standard deviation /40)	31.9 ± 5	32.7 ± 5.1	0.01
Fatigue FACIT-F^a^ total scale, (mean ± standard deviation /160)	118 ± 24	125 ± 22	< 0.0001
Pain analogic scale, (mean ± standard deviation /10)	1.4 ± 1.9	1.1 ± 1.8	0.004

Results of the self-esteem, fatigue and pain scales, among participants with sport activity (N = 550) vs without activity (N = 610). A higher score is associated, respectively with higher self-esteem, higher pain and lower fatigue

^a^FACIT-F: Functional Assessment of Chronic Illness Therapy Fatigue

## Discussion

The proportion of patients having a sport activity is 47% in our study: this is slightly more than in the general adult population of our region: 43% in 2017 [7].

The self-esteem mean score was 32.2/40 in our population: it represents a medium self-esteem. This score is slightly better than that of a study with 253 adults of the general French population: Rosenberg self-esteem mean score: 30.9 [8].

Sport activity is associated with better self-esteem in our study. This is concordant with a meta-analysis of studies in the general adolescent population, which shows that physical activity intervention improves feelings of self-worth [9]. In the adult population, some randomized studies show a beneficial impact of sport on self-esteem. In a Spanish randomized study of 360 adults with chronic diseases, self-esteem increased significantly in the walking program group [10]. In the PLHIV population, a randomized study with 40 men showed that a twice-weekly, 6-month sport program, improved self-efficacy [3].

Fatigue was lower among sporting people in our study. Similarly, Webel and al. [11] found that lower fatigue was associated with physical activity, among 5300 PLHIV in the USA. Similar results have been shown in cancer patients. In a review of 56 randomized studies that investigated the effect of exercise on cancer-related fatigue, aerobic exercise helped to reduce fatigue both during and after treatment of cancer [12].

Pain was lower among sporting people in our study. Similarly, lower pain was associated with physical activity in the study of Webel and al. [11]. In a randomized study of 33 cancer patients receiving chemotherapy, pain was significantly reduced in the sporting group (biking) [13]. Possible explanations are activation of central pain inhibitory systems and higher production of endorphins [14].

Our study has limitations. Subjects with difficulties in understanding French were likely under-represented. To avoid confounds, multivariable analyses are more relevant. Our results cannot be extrapolated to the general population living with HIV. However, the strength of our study is the significant size of the PLHIV population.

## Conclusions

Among PLHIV in our region, sport activity was associated with better self-esteem, lower fatigue and lower pain. Thus, health care workers should encourage PLHIV to perform sport activity.

## Data Availability

The data-sets used and/or analysed during the current study are available from the corresponding author on reasonable request.

## Abbreviations

ART: Anti-retroviral therapy
BMI: Body mass index
CD4: Cluster of differentiation 4
CDC: Centers for Disease Control
FACIT-F: Functional Assessment of Chronic Illness Therapy-Fatigue
HCV: Hepatitis C infection
HBV: Hepatitis B infection
HIV: Human immunodeficiency virus
IQR: Inter-quartile range
MSM: Men who have sex with men
PLHIV: People living with human immunodeficiency virus

## References

[1] O'Brien KK, Tynan AM, Nixon SA, Glazier RH (2016). Effectiveness of aerobic exercise for adults living with HIV: systematic review and meta-analysis using the Cochrane Collaboration protocol. BMC Infect Dis.

[2] O'Brien KK, Tynan AM, Nixon SA, Glazier RH (2017). Effectiveness of Progressive Resistive Exercise (PRE) in the context of HIV: systematic review and meta-analysis using the Cochrane Collaboration protocol. BMC Infect Dis.

[3] Fillipas S, Oldmeadow LB, Bailey MJ, Cherry CL (2006). A six-month, supervised, aerobic and resistance exercise program improves self-efficacy in people with human immunodeficiency virus: a randomised controlled trial. Aust J Physiother.

[4] Ibeneme SC, Uwakwe V, Myezwa H (2022). Impact of exercise training on symptoms of depression, physical activity level and social participation in people living with HIV/AIDS: a systematic review and meta-analysis. BMC Infect Dis.

[5] Juth V, Smyth JM, Santuzzi AM (2008). How do you feel? Self-esteem predicts affect, stress, social interaction, and symptom severity during daily life in patients with chronic illness. J Health Psychol.

[6] Mills EJ, Nachega JB, Bangsberg DR, Singh S, Rachlis B, Wu P (2006). Adherence to HAART: a systematic review of developed and developing nation patient-reported barriers and facilitators. PLoS Med..

[7] Observatoire Régional de la Santé des Pays de la Loire (2019): Statut pondéral, activité physique, pratique sportive dans les Pays de la Loire. Résultats du Baromètre de Santé publique France 2017, https://www.orspaysdelaloire.com/sites/default/files/pages/pdf/2019_PDF/2019_bs2017_statutponderal_1.pdf. Accessed 01 May 2022.

[8] Petit F, Hubert-Buron A, Mollet-Boudjemline A, Sechepine A, Milcent K, Guyonnet C (2013). Mise au point et validation d'un outil d'évaluation de la santé sexuelle sous forme d'auto-questionnaires pour une application aux maladies métaboliques [Construction and validation of an evaluation tool of sexual health using self-administered questionnaires for an application to metabolic diseases]. Prog Urol.

[9] Liu M, Wu L, Ming Q (2015). How does physical activity intervention improve self-esteem and self-concept in children and adolescents? Evidence from a meta-analysis. PLoS ONE.

[10] Villalobos F, Vinuesa A, Pedret R, Reche A, Domínguez E, Arija V (2019). Efecto de un Programa de actividad física sobre la autoestima en sujetos con enfermedades crónicas. Ensayo de intervención comunitaria «Pas a Pas» [Effect of a Physical activity program on self-esteem in subjects with chronic diseases]. 'Pas a Pas' community intervention trial. Aten Primaria..

[11] Webel AR, Willig AL, Liu W, Sattar A, Boswell S, Crane HM (2019). Physical activity intensity is associated with symptom distress in the CNICS Cohort. AIDS Behav.

[12] Cramp F (2012). Exercise for the management of cancer-related fatigue in adults. Cochrane Database Syst Rev..

[13] Dimeo F, Fetscher S, Lange W, Mertelsmann R, Keul J (1997). Effects of aerobic exercise on the physical performance and incidence of treatment-related complications after high-dose chemotherapy. Blood.

[14] Droste C (1992). Transient hypoalgesia under physical exercise-relation to silent ischaemia and implications for cardiac rehabilitation. Ann Acad Med Singap.

